# TwinSpectra—Spectroscopic scans of refuse-derived fuel particles

**DOI:** 10.1016/j.dib.2026.112920

**Published:** 2026-06-02

**Authors:** Jonas Fischer, Mina Bikhit, Łukasz Wrześniowski, Adriana Niepala, Jakub Mielcarek, Enric Illana-Mahiques, Bogdan Ruszczak

**Affiliations:** a Ruhr-Universität Bochum, Universitätsstraße 150, D-44780 Bochum, Germany; b Opole University of Technology, Prószkowska 76 Street, 45-758 Opole, Poland

**Keywords:** Dataset, Spectroscopic scans, Waste classification, Sustainable manufacturing, Machine learning

## Abstract

TwinSpectra is a novel data comprising spectroscopic measurements of refuse-derived fuel particles sampled during the cement manufacturing process. The data consists of two alternative sets of material reflectance spectra acquired using two spectroradiometers based on distinct measurement principles. In total, 120 samples representing six particle groups are contained in the datasets, with each sample measured twice using each device. After data preprocessing and cleansing, two separate files of 718 and 720 measurements were compiled, respectively, for a total of 1438 readouts. The whole process was carried out with respect to the required calibration procedures. Eventually, all the records were assigned to one of six material groups that could be later utilized as classes. This data can serve as a solid foundation for subsequent analyses and the development of machine learning-based solutions. In particular, the datasets and the algorithms built on them should enable the automation of sustainable cement manufacturing or aid in the development of techniques for working with highly dimensional, sequential spectroscopic data.

Specifications Table

**Table:** 

Subject	Computer Sciences
Specific subject area	Two sets of spectroscopic reflectance measurements of various materials with class labels.
Type of data	2 tables (with spectroscopic measurements and class labels) stored in CSV format. Filtered (measurements acquired using calibrated devices and compiled into tidy data tables with erroneous samples filtered out).
Data collection	The data is built of two sets of spectroscopic scans of various particles that are being sampled during the cement manufacturing process. As this process is dynamic, a reliable and automated material detection method is highly sought after. Thus, we prepared this collection to enable the development of remote sensing detection algorithms. Two sets were acquired using two spectroradiometers, which allow for high resolution spectra registration, with slightly different acquisition processes. These were: the ASD FieldSpec 4 Hi-Res, with the working range of 350 - 2500 nm and 2151 consecutive spectra readouts, and Viavi microNIR, which returns 125 readouts in the range of 908.1 - 1676.2 nm.
Data source location	- Ruhr-Universität Bochum Bochum, Germany, N 51° 26.665 E 7° 15.678 - Opole University of Technology Opole, Poland, N 50° 39.153 E 17° 54.333
Data accessibility	Repository name: TwinSpectra Dataset [1] Data identification number: DOI: 10.5281/zenodo.18471661 Direct URL to data: https://zenodo.org/records/18471661
Related research article	[[8], [9], [10]]

## 1 Value of the Data

• Unique collection of high spectral resolution measurements, with one of six material groups assigned to each sample.
• Machine-learning-ready dataset suitable for multi-class classifiers.
• Two measurement processes using distinct spectroradiometers were employed, enabling the evaluation of multimodal algorithms.
• The provided high-resolution scans allow for the discovery of useful spectral parts and the development of algorithms that support comprehensive classifier explainability.
• The dataset is suitable for dimensionality reduction techniques of multidimensional datasets [2] or development [3] and optimization [4] of preprocessing techniques for spectroscopic data.
• A solid data source for supporting a method for automation of sustainable cement manufacturing with refuse derived fuel combustion.
• This data might be valuable for:
  ○ Researcher in the area of spectroscopy.
  ○ Researcher in the area of machine learning and computer vision. especially classification and super resolution.
  ○ Researcher in the area of recycling technology.
  ○ Developer of automation solution for cement and recycling industry.

## 2 Background

Cement manufacturing is an energy-intensive industrial process, traditionally reliant on fossil fuel combustion. Beyond the inherent CO_2_ released during calcination of limestone, fuel combustion accounts for approximately 40 % of cement production’s emissions [5]. Transitioning to Refuse-Derived Fuel (RDF), which is derived from municipal and industrial waste, presents a dual opportunity: reduce both the amount of non-recyclable waste and CO_2_ emissions, as RDF combustion emits 50 g CO_2_/MJ [6] while coal combustion emits 94 g CO_2_/MJ [7].

Due to its origin from wastes, composition of RDF is also heterogeneous over time and production plants. This volatility makes consistently high-quality cement production with high RDF fuel replacement rate a difficult task. Some material groups are nonetheless common in most RDFs: Foils, 3D plastics, paper, cardboard, foams, textiles and rubber. These material groups differ in characteristic properties like heating value, flight behavior or moisture [8], which makes the RDF composition an important parameter of kiln operation.

Currently, the lack of automated, real-time quality control in production plants prevents precise fuel characterization. Approaches based on images [9] and NIRS [10] have been developed but are not yet used in industry. The approaches rely on single particles and their images/spectra. Problems for the adaption are overlapping particles, sensor dust contamination and changing fuel streams (drift from training data). With images, no information about plastic types can be extracted, which are especially important considering the chlorine content of PVC. This data gap is the primary barrier to optimizing RDF integration in cement production kilns.

## 3 Data Description

The paper delivers two collections of spectroscopic measurements, which are acquired using two distinct devices. Those measurements are stored in two CSV files:

• “measurements_2151.csv” –reflectance measurements in range **from 350 to 2,500 nm.** The reflectance is measured with 1 nm step, and the number of columns with reflectance values is **2151**. Additional data on scanned particle group, sample subset (folds 0 to 4), sample name and id is stored.
• “measurements_125.csv” –reflectance measurements in range from **908.1 to 1,676.2 nm**. It holds **125** columns of reflectance values with steps of approximately 6.2 nm. Additional data is encoded in accordance with the other file, with same configuration of groups and folds.

A detailed information on the content of both dataset files is provided in Table 1. The first column, called *Parameter*, identifies the attribute names. The following columns provide the content description, and the content of both dataset files.

**Table 1:** The additional metadata for each measurement encoding that is maintained for both CSV dataset files.

Parameter	Description	measurements_2151	measurements_125
id	The source particle identification number	302, 305, 307, …, 1826, 1828, 1834.	302, 305, 307, …, 1826, 1828, 1834.
sample	The following source scan file identification number	9, 10, 11, …, 775, 776, 777.	0, 1, 2, …, 717, 718, 719.
group	One of the six particle groups	3D, Foam, Foil, PC, Rubber, Textile	3D, Foam, Foil, PC, Rubber, Textile
fold	Identification of five subsets for the cross-validation procedure for the machine learning models evaluation	0, 1, 2, 3, 4	0, 1, 2, 3, 4

In Table 2, the distribution of measurements for refuse-derived fuel particles across distinct groups and subfolds is presented. For both files, we randomly and independently split the readouts into 5 folds, enabling the eventual evaluation of a credible machine learning algorithm. We used bold text in Table 2 to highlight the numbers summarizing the column-wise and row-wise aggregations of measurements. This allows to verify the balance of samples across groups and subfolds.

**Table 2:** The distribution of measurements among particle groups and folds. The bold numbers represent accumulated count for folds (columns) and particle groups (rows).

group	measurements_2151						measurements_125					
		fold						fold				
	Σ	0	1	2	3	4	Σ	0	1	2	3	4
**3D**	**120**	24	24	24	24	24	**120**	24	24	24	24	24
**Foam**	**120**	24	24	24	24	24	**120**	24	24	24	24	24
**Foil**	**120**	24	24	24	24	24	**120**	24	24	24	24	24
**PC**	**120**	24	24	24	24	24	**120**	24	24	24	24	24
**Rubber**	**120**	24	24	24	24	24	**120**	24	24	24	24	24
**Textile**	**118**	24	24	24	23	23	**120**	24	24	24	24	24
Σ	**718**	**144**	**144**	**144**	**143**	**143**	**720**	**144**	**144**	**144**	**144**	**144**

While acquiring the scans for *measurements_2151*, we identified one of the Textile particles as being too small to scan precisely with the ASD FieldSpec. Therefore, there are two fewer samples for this group (every other particle has been scanned twice), and one sample less for the last two folds of this subset.

In Table 3, Table 4 we present randomly selected examples from both dataset files (measurements_2151.csv and measurements_125.csv, respectively). Because the files contain a large number of columns, we only present the first and the last three columns of spectroscopic readouts.

**Table 3:** Selected samples from the *measurements_2151.csv* dataset file.

id	sample	group	fold	350.0	351.0	352.0	…	2498.0	2499.0	2500.0
1648	226	Rubber	0	0.1723	0.1663	0.1693		0.0838	0.0841	0.0839
1142	329	Foam	1	0.0338	0.0342	0.0315		0.0517	0.0512	0.0512
541	438	PC	2	0.1184	0.1106	0.1053		0.2517	0.2516	0.2519
341	541	Foil	3	0.1736	0.1715	0.1692	…	0.1053	0.1060	0.1060
1444	122	Textile	4	0.0513	0.0490	0.0423		0.0621	0.0622	0.0621
370	652	3D	1	0.1766	0.1715	0.1760		0.0472	0.0467	0.0457

**Table 4:** Selected samples from the *measurements_125.csv* dataset file.

id	sample	group	fold	908.10	914.29	920.49	…	1663.81	1670.01	1676.20
302	0	PC	0	0.3530	0.3575	0.3629		0.3892	0.3890	0.3887
311	5	3D	1	0.2722	0.2765	0.2826		0.2836	0.2925	0.3014
356	32	Foil	2	0.4061	0.4049	0.4036		0.5038	0.5044	0.5039
305	1	Textile	3	0.2946	0.2961	0.2993	…	0.2885	0.2901	0.2922
456	449	Foam	4	0.4916	0.5038	0.5175		0.5674	0.5711	0.5731
379	402	Rubber	2	0.2740	0.2649	0.2569		0.3343	0.3395	0.3466

**Fig. 1:**
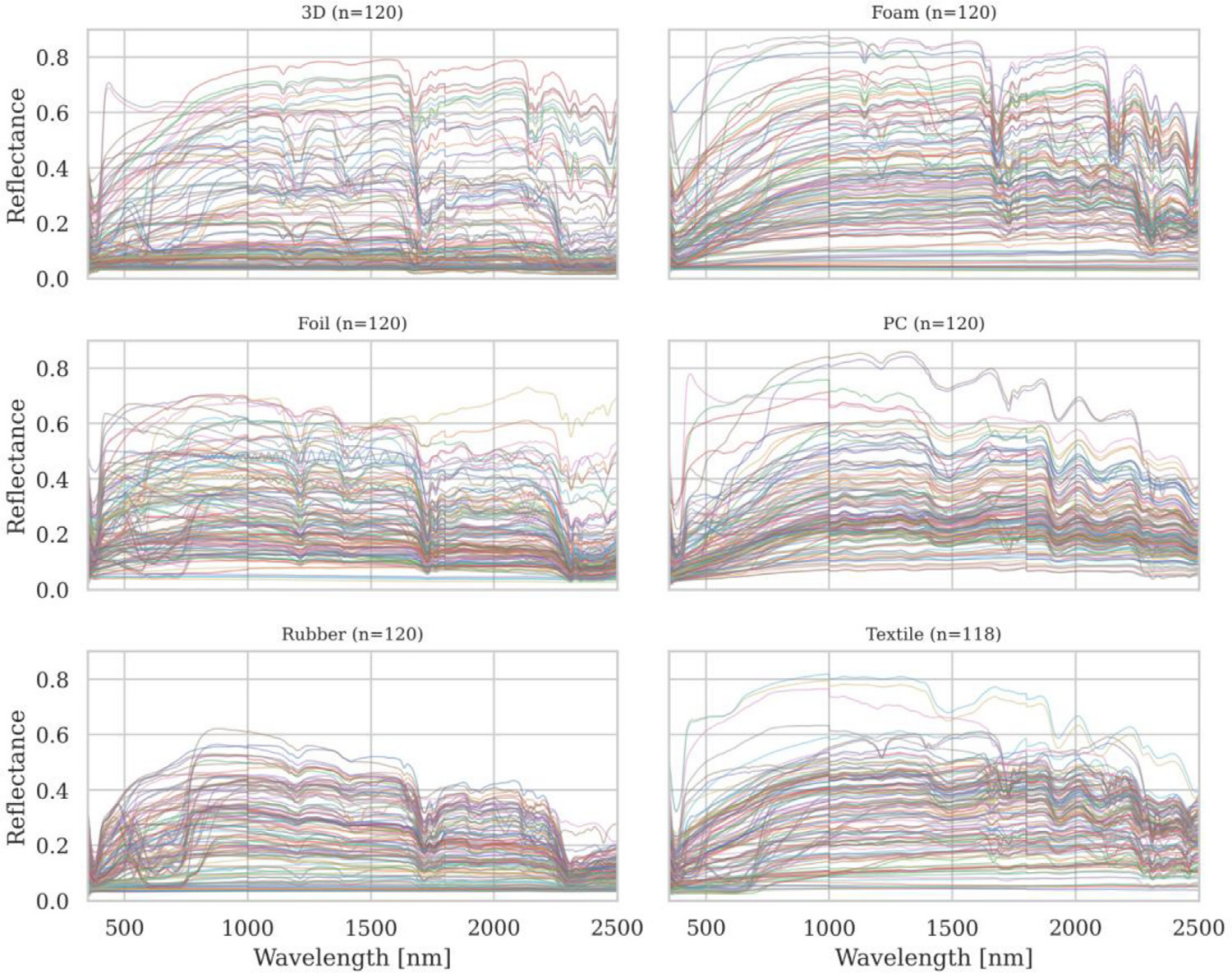
The measurements visualization for the dataset with 2151 spectral features for the 350 to 2500 nm spectral range with the average spectral resolution of 1 nm. For the 6 distinct classes we depict the measurements of consecutive scanned samples.

**Fig. 2:**
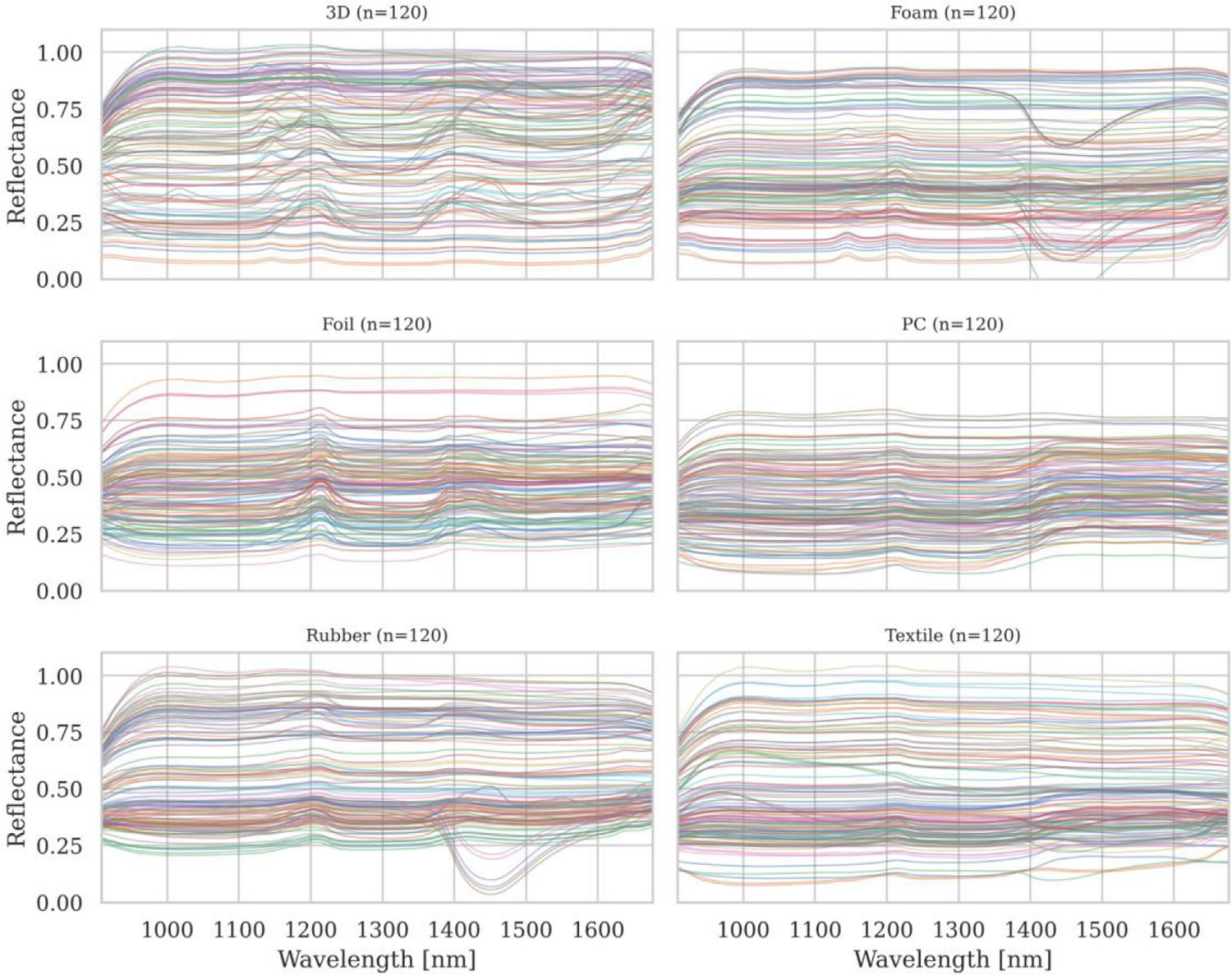
The measurements visualization for the dataset with 125 spectral features for the 908.1 to 1676.2 nm spectral range, with the average spectral resolution of 6.2 nm. For the 6 distinct classes we depict the measurements of consecutive scanned samples.

All measurements collected using the ASD FieldSpec 4 Hi-Res spectrophotometer are depicted on Fig. 1 and for those taken with the Viavi microNIR device on Fig. 2.

## 4 Experimental Design, Materials and Methods

### 4.1 The refuse-derived fuel particles selection

The refuse derived fuels were sampled in eight German cement plants by operating staff of the cement producers [11]. To ensure biological stability, the moisture was removed by oven-drying such that all samples are dry. Fine particles were subsequently removed by sieving with a 8 mm sieve. The remaining material was categorized into six distinct groups following [10], an updated classification framework based on [12]. The sorting was executed as following:

1. Manual sieving with an 8 mm sieve. Remove and weigh fine parts, this is too small to be sorted or measured by NIR. Fine particles seem to be a mix of all fractions can make up around 10-30 % of the total RDF mass.
2. Review each particle individual by optical and haptic assessments and assign one fraction (Rubber, Foam, Paper and Cardboard, Textile, Foils, 3D plastics) to it. If the particle is a plastic particle, an additional differentiation is made: If the particle is thin and flexible, it is assigned to foils, if it is rigid and bulky, it is considered a 3D plastic. Particles not belonging to one of the six fractions are collected separately and weighed after sorting. These particles can be non-ferrous metal (iron is magnetically separated in cement plants), glass or stone, but make up <1 %.
3. Assign each particle an individual number PXXX.

While some literature, such as [13], identifies up to 12 material groups, these often include components like glass or metal that are rarely present in processed RDF. The composition varied between sites, though thin foils, paper, cardboard, and rigid 3D plastics remained the dominant constituents.

**Fig. 3:**
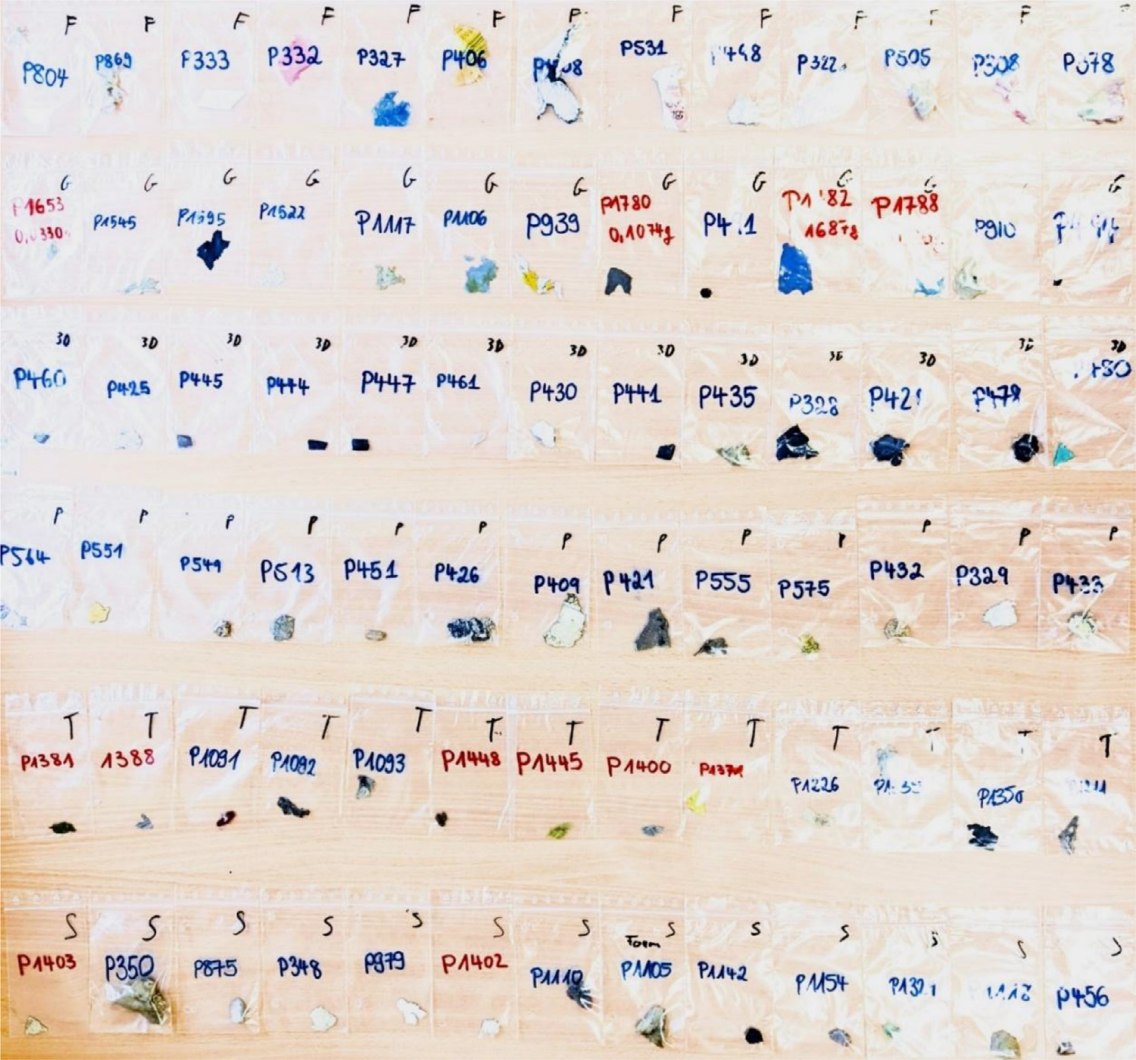
Images (around 20 % of the whole dataset depicted) of the scanned refuse-derived fuel particles from each of the six different particle groups. Each particle was individually packaged with its ID and group indicated.

60 particles were selected from each material group, resulting in a total of 360 particles. Part of the samples are displayed on Fig. 3. Detailed descriptions of each material group follow:

• Foils: Comprising the largest fraction of RDF (approximately 35 % by weight), these thin (< 1 mm) flexible plastic particles are highly desirable. Their high heating value (31 MJ/kg) and favorable flight behavior [14] – driven by a high surface-area-to-mass ratio – ensure complete combustion before reaching the clinker bed.
• 3D plastics: This category contains all non-foil plastics. These rigid particles have a lower surface-area-to-mass ratio and a high heating value (37 MJ/kg). However, their poor aerodynamic flight behavior often results in incomplete incineration and unburnt particles reaching the clinker bed, which can induce unwanted reducing conditions.
• Paper and Cardboard (PC): A relatively homogeneous group of flat particles (around 1.5 mm thick). While their heating value is lower (17 MJ/kg), they are largely considered CO_2_ neutral. Notably, PC acts as the primary moisture sink within RDF mixtures.
• Textiles: Primarily composed of fiber bundles or fabric, textiles often include synthetic polymers, cotton (biogenic content) and tire cord (rubber-integrated). These fibers frequently trap fine particles from other groups. Their heating value averages 22 MJ/kg. A commonly used fiber is tire cord, such that some parts of the textiles may contain rubber.
• Foams: Consisting of both polystyrene and polyurethane, foams have a heating value of 27 MJ/kg. Despite their spherical morphology, their low density ensures flight behavior that does not compromise cement quality.
• Rubber: Rubber are flexible elastomer particles, mostly from industrial waste, e.g., hoses or gaskets. The heating value is 28 MJ/kg. Rubber particles are not as common as the other material groups. Often their color is black, which makes an online measurement on a rubber material belt challenging.

## 5 Employed Spectroradiometers and the Measurement Procedure

The monitoring process of the manufacturing process could vary, and more frequently it is carried out using regular or multispectral cameras or sensors. In this study, to enable further investigation, we employed hyperspectral spectroradiometers to provide high-resolution readouts of the refuse-derived fuel particles used in the cement manufacturing process. The detailed information of the scanned particles could help to develop better monitoring methods. We used two distinct devices to provide a broader view of the scanned phenomena. One with very broad recorded spectra and another one with focus on the near-infrared range. In addition, the two devices differ substantially in their price range, with the ASD FieldSpec 4 Hi-Res representing the higher-cost instrument.

The first spectroradiometer used was the ASD FieldSpec 4 Hi-Res. For the presented dataset, we employed this device to acquire the reflectance of the materials, although it also supports transmission, radiance or irradiance registration. The working range for scanning the samples is 350-2500 nm. The spectral sampling width was 1.4 nm for the UV-VNIR range (350-1000 nm) and 1.1 nm for the VNIR-SWIR range (1001-2500 nm). This spectroradiometer is built of 3 detectors [15]: one 512-element silicon near-infrared sensor (VNIR: 350-1000 nm), and two InGaAs photodiode-based, 2 Stage TE Cooled Graded detectors (SWIR_1_: 1000-1800 nm and SWIR_2_: 1800-2500 nm). For the measurement process following the advised calibration procedure the recorded wavelength reproducibility of 0.1 nm is guaranteed, with a wavelength accuracy of 0.5 nm for the average error of wavelength calibration fit, the wavelength accuracy of ±1 nm for any other line, and the Noise Equivalent Radiance of: 1.0·10^-9^ W/cm^2^/nm/sr, 1.4·10^-9^ W/cm^2^/nm/sr, 2.2·10^-9^ W/cm^2^/nm/sr, for detectors: VNIR at 700 nm, SWIR_1_ at 1400 nm and SWIR_2_ at 2100 nm respectively [15]. During all measurements, we maintained the same scan height and lightning position and carefully followed the dark-current and white-reference calibration procedures.

The second employed device was a Viavi microNIR. It is a small (47 mm by 60 mm), mobile and USB-powered device. The device includes one 128 pixel InGaAs photodiode sensor and two tungsten lamps. The intended working mode is reflectance, which was therefore used for this dataset. The working range is 908-1676 nm with a resolution of 6.2 nm, resulting in 125 data points per measurement. The wavelength accuracy is < 3 nm with a repeatability of < 1 nm [16]. Before the measurement and after every 15 min, the dark-current and white-reference calibration was renewed. The measurement distance was fixed by a fixed attachment.

## 6 Limitations

• The employed devices have higher spectral resolution than popular manufacturing monitoring sensors, and additional effort is required to translate the results into a method that would be production-ready.
• The limited field of view of the applied devices and the small size of some samples introduce additional noise into the measurements.
• The readouts from two different spectroradiometers do not directly translate to each other, and research towards benefiting from having them both is required.
• To avoid the influence of the background, a pure black material was used while scanning the samples. However, in the actual manufacturing process, the background could vary and the spectral signatures of the doped composites could differ.
• For deep learning applications, the amount of data may be too small and too ideal (lower noise than in real world systems). Therefore, data augmentation like shifting the baseline, mixing of data of the same fraction or adding gaussian noise. Additionally, changes in preprocessing algorithms like standard normative variate or multiplicative scatter correction can be used to artificially increase the data size.

## Acronyms

**Table:** 

Acronym	Meaning
NIR	Near-InfraRed spectroscopy
RDF	Refuse Derived Fuel
VNIR	Visible and Near-InfraRed
SWIR	Short Wave InfraRed
PC	Paper and Cardboard
InGaAs	INdium GAllium ArSenide

## Ethics Statement

The authors have read and follow the ethical requirements for publication in Data in Brief and confirming that the current work does not involve human subjects, animal experiments, or any data collected from social media platforms.

## Credit Author Statement

**Jonas Fisher:** Conceptualization, Methodology, Software, Validation, Investigation, Resources, Data Curation, Writing - Original Draft, Writing - Review & Editing, Funding acquisition; **Łukasz Wrześniowski:** Validation, Investigation, Writing - Review & Editing, Visualization; **Mina Bikhit:** Validation, Investigation, Writing - Review & Editing; **Jakub Mielcarek:** Validation, Investigation, Writing - Review & Editing; **Adriana Niepala:** Validation, Investigation, Writing - Review & Editing; **Enric Illana-Mahiques:** Writing - Review & Editing; **Bogdan Ruszczak:** Conceptualization, Methodology, Software, Validation, Investigation, Resources, Data Curation, Writing - Original Draft, Writing - Review & Editing, Visualization, Supervision.

## Data Availability

ZenodoTwinSpectra Dataset (Original data).
